# Protopanaxadiol improves endometriosis associated infertility and miscarriage in sex hormones receptors-dependent and independent manners

**DOI:** 10.7150/ijbs.58657

**Published:** 2021-05-05

**Authors:** Zhen-Zhen Lai, Hui-Li Yang, Jia-Wei Shi, Hui-Hui Shen, Yan Wang, Kai-Kai Chang, Tao Zhang, Jiang-Feng Ye, Jian-Song Sun, Xue-Min Qiu, Ming-Qing Li

**Affiliations:** 1 NHC Key Lab of Reproduction Regulation (Shanghai Institute of Planned Parenthood Research), Hospital of Obstetrics and Gynecology, Shanghai Medical School, Fudan University, Shanghai 200080, People's Republic of China.; 2 Laboratory for Reproductive Immunology, Hospital of Obstetrics and Gynecology, Shanghai Medical School, Fudan University, Shanghai 200080, People's Republic of China.; 3 Department of Gynecology, Hospital of Obstetrics and Gynecology, Shanghai Medical School, Fudan University, Shanghai 200011, People's Republic of China.; 4 Assisted Reproductive Technology Unit, Department of Obstetrics and Gynecology, Faculty of Medicine, Chinese University of Hong Kong, Hong Kong, People's Republic of China.; 5 Division of Obstetrics and Gynecology, KK Women's and Children's Hospital, 229899, Singapore.; 6 National Research Centre for Carbohydrate Synthesis, Jiangxi Normal University, 330022 Jiangxi, Nanchang, China.; 7 Shanghai Key Laboratory of Female Reproductive Endocrine Related Diseases, Hospital of Obstetrics and Gynecology, Shanghai Medical School, Fudan University, Shanghai, 200080, People's Republic of China.

**Keywords:** endometriosis, protopanaxadiol, endometrial receptivity, decidualization, GnRHa

## Abstract

**Background:** Patients with endometriosis (EMs) have high risks of infertility and spontaneous abortion. How to remodel the fertility of patients with EMs has always been the hot spot and difficulty in the field of reproductive medicine. As an aglycone of ginsenosides, protopanaxadiol (PPD) possesses pleiotropic biological functions and has high medicinal values. We aimed to investigate the effect and potential mechanism of PPD in the treatment of EMs-associated infertility and spontaneous abortion.

**Methods:** The EMs mice models were constructed by allotransplantation. The pregnancy rates, embryo implantation numbers and embryo resorption rates of control and EMs were counted. RNA sequencing, qRT-PCR, enzyme linked immunosorbent assay (ELISA) and FCM analysis were performed to screen and confirm the expression of endometrial receptivity/decidualization-related molecules, inflammation cytokines and NK cell function-related molecules *in vitro* and/or *in vivo*. The SWISS Target Prediction, STRING and Cytoscape were carried out to predict the potential cellular sensory proteins, the protein-protein interaction (PPI) network between sensory proteins and fertility-related molecules, respectively. Micro-CT detection, liver and kidney function tests were used to evaluate the safety.

**Results:** Here, we observe that PPD significantly up-regulates endometrial receptivity-related molecules (e.g.,* Lif, Igfbp1, Mmps, collagens*) and restricts pelvic inflammatory response (low levels of IL-12 and IFN-γ) of macrophage, and further remodel and improve the fertility of EMs mice. Additionally, PPD increases the expression of decidualization-related genes and *Collagens*, and promotes the proliferation, residence, immune tolerance and anagogic functions of decidual NK cells (low levels of CD16 and NKp30, high levels of Ki67, VEGF, TGF-β) in pregnant EMs mice, and further triggers decidualization, decidual NK cell-mediated maternal-fetal immune tolerance and angiogenesis, preventing pregnant EMs mice from miscarriage. Mechanically, these effects should be dependent on ESRs, PGR and other sensory proteins (e.g., AR). Compared with GnRHa (the clinic first-line drug for EMs), PPD does not lead to the decline of serum estrogen and bone loss.

**Conclusion:** These data suggest that PPD prevents EMs-associated infertility and miscarriage in sex hormones receptors-dependent and independent manners possibly, and provides a potential therapeutic strategy with high efficiency and low side effects to remodels the fertility of patients with EMs.

## Introduction

Endometriosis (EMs) is a common, chronic gynecological disease which can happen in women of reproductive age 1. The prevalence of EMs approximately to be 5%~10% 2, with a peak from 25 years old to 35 years old 3, and up to 50% of infertile women 4. EMs, an estrogen-dependent chronic inflammatory disease, is associated with pelvic pain and infertility 5. Notedly, in spontaneous pregnancies, endometriosis appears to be a risk factor of miscarriages (almost 80% increased risk 6). There were varieties of epidemiological factors and molecular and cellular alterations of EMs had been reported, such as early age at menarche or a long duration of menstrual flows 7, familial aggregation 8, increased estrogen receptor (ER) α and β expression 9-11, progesterone resistance 9, overproduction of prostaglandins, cytokines and chemokines 12, 13, and so on.

The reasons for EMs-associated infertility and miscarriage have been completely unknow yet. Pelvic anatomy distortion, endocrine and ovulatory disorders, inflammation and immune disorders, reduced endometrial receptivity, dysfunctional fallopian tubes and impaired sperm transport are the most common reasons for EMs-associated infertility 14. Macrophages (Mφ) in the abdominal cavity are the main source of cytokines that are either involved in regulating inflammation or released upon injury to the peritoneum 15, 16. Mφ constitutes 50% of peritoneal leukocytes in human. Dysfunctional phenotypes of peritoneal Mφ were observed in women with EMs, characterized by reducing phagocytic capacity and producing more Prostaglandin E2 (PGE_2_) and a lot of proinflammatory cytokines, which contribute to pelvic inflammation and anatomical abnormalities 17, 18.

Endometrial receptivity refers to the ability of the maternal endometrium to accept embryo implantation at a specific time. This period is called the “implantation window period” 19, which is regulated by estrogen and progesterone. These changings of sex hormones prevent menstruation, destruction of the decidualized endometrium, regulate lymphocytes functions, and promote angiogenesis. In the early pregnancy, the accumulation of leukocytes is up to 40% of total decidual cells, and decidual NK (dNK) cell population accounts for about 70% of total tissue immune cells 20, 21. dNK cells, marked by CD56^bright^CD16^-^KIR^+^, are poorly cytotoxic and have positive effects in regulating decidualization, placental development and angiogenesis 22, 23. Currently, researches on the role of dNK cells in endometrium of EMs patients are still scarce. However, more and more studies have shown the decreased number and activated cytotoxicity of dNK are closed related to spontaneous abortion 24-26.

Although the current medical therapies (e.g., gonadotropin-releasing hormone agonists (GnRHa), progestins and aromatase inhibitors) are world wildly used to inhibit ectopic endometrium growth by reducing the systemic levels of estrogen, their side effects cannot be ignored 9. Therefore, the most urgent need is to develop more effective treatments and to save the fertility of patients to the greatest extent. Nowadays, traditional Chinese medicine has also been scientifically proven to be effective 27. Ginseng is one of the most famous traditional medicinal herbs, which is widely used in Asian countries. Protopanaxadiol (PPD) is one of two metabolites of ginsenoside, the main components extracted from ginseng. There are many reports about PPD used to treat various tumors, cardiovascular diseases 28, 29. Additionally, our previous study has showed that PPD suppresses ER-mediated inhibition of endometriotic cells autophagy, contributing to anti-EMs effects 30. However, the possible role and mechanism for PPD on infertility and spontaneous abortion of EMs are unknown.

Therefore, the current study is to investigate whether and how PPD against on infertility and spontaneous abortion of EMs *in vitro* and *in vivo*, and provide the potential intervention strategies for fertility remodeling and preservation of patients with EMs.

## Materials and Methods

### EMs mice model

A group of adult female BALB/C mice aged 6-8 weeks was purchased from Shanghai Jiesijie Experimental Animal Co., Ltd. (China) and was used for this study. They were maintained for 2 weeks at the animal facility before use. The Animal Care and Use Committee of Fudan University approved all the animal protocols. We constructed an intraperitoneal EMs model. On day 0, one-third of all mice were randomly selected as donors, and their uterus horns were removed and cut into fragments smaller than 1 mm^3^. Then those fragments were suspended in sterile saline and injected them into the remains intraperitoneally (for recipient mice, the ratio of the uterus to intraperitoneal injection of mice was 1:2). On the day 4, peritoneal fluids and uterus of Ctrl and/or EMs mice were collected, or the rest of Ctrl and/or EMs mice were randomly mated with healthy male adult mice, respectively, and then the data of pregnancy rate, the number of embryos, and the absorption rate were counted on day 18. From day 4 to day 32, the recipient mice were randomly divided into 3 groups and injected intraperitoneally with vehicle control (0.5% DMSO, every day, Sigma, MA, USA) or 45 mg/kg PPD (every 4 days, Sigma, MA, USA), or injected intramuscularly with 0.5μg GnRHa (every day, Sigma, MA, USA). The dose of PPD at 45 mg/kg was based on our previous study 30, and the dose of GnRHa at 0.5 μg was modified from a previous research conducted in female rats 31. On day 32, half of the mice were selected to mate with healthy male adult mice, and then the tissues of uterus were collected, and the data of pregnancy rate, the number of embryos, and the absorption rate were counted on day 46. In the remaining mice, the serum, the EMs-like lesions, peritoneal fluids (PF), uterus, livers, kidneys, and femurs were collected on day 32. The levels of estradiol in the serum were detected by enzyme linked immunosorbent assay (ELISA). The levels of aspartate aminotransferase (AST) and blood urea nitrogen (BUN) in the serum were measured by an automatic biochemical analyzer (Beckman Counter, USA). The serum levels of tartrate resistant acid phosphatase (TRAP) and alkaline phosphatase (ALP) were measured. Micro-CT was used to measure the osteoporosis-related markers. The number and weight of EMs-like lesions were counted. Hematoxylin-Eosin (H&E) staining was used to assess the lymphocytes infiltration in the livers and kidneys.

### Flow cytometry (FCM) analysis

Human and mouse antibodies for flow cytometry assays (all antibodies were purchased Biolegend, CA, USA) were used for measurement of cell markers, including APC/Cyanine 7 (APC/Cy7)-conjugated anti-mouse CD45, fluorescein isothiocyanate (FITC)-conjugated anti-mouse F4/80, brilliant violent (BV) 660-conjugated anti-mouse CD11b, BV510-conjugated anti-mouse interferon (IFN)-γ, APC-conjugated anti-mouse interleukin (IL)-12, FITC-conjugated anti-mouse CD3, PE-Cy7-conjugated anti-mouse CD49a, BV510-conjugated anti-human CD14, BV421-conjugated anti-human CD45, APC/Cy7-conjugated anti-human CD45, phycoerythrin/Cyanine 7 (PE/Cy7)-conjugated anti-human IFN-γ, FITC-conjugated anti-human IL-12, BV605-conjugated anti-human CD56, PE/Cy7-conjugated anti-human CD16, PE-conjugated anti-human NKp30, APC-conjugated anti-human Ki67, APC-conjugated anti-human CXC chemokine ligand (CXCL)10, APC/Cy7-conjugated anti-human vascular endothelial growth factor (VEGF), and BV421-conjugated anti-human transforming growth factor (TGF)-β. Isotype IgG antibody (5 μl separately) was used as the control. Human Trustain FcX (Biolegend, CA, USA) was used to block Fc receptors prior to flow cytometry. Subsequently, cells were washed twice and resuspended in PBS for flow cytometry analysis. Samples were analyzed using a CytoFLEX flow cytometer (Beckman Coulter, Inc.) and data were analyzed using FlowJo (version 10.07 (FlowJo LLC).

### Quantitative Real-Time Polymerase Chain Reaction (qRT-PCR)

The total RNA was extracted by TRIzol regent (Invitrogen, Carlsbad, CA, USA). Subsequently, the concentration and purity of RNA was quantified by a NanoDrop spectrophotometer (NanoDrop Technologies; Thermo Fisher Scientific, MA, USA). The PrimeScript RT Reagent Kit (TaKaRa Biotechnology, Co., Ltd., Dalian, China) was utilized to reversely transcribe total RNA to cDNA. Next, qRT-qPCR was performed with SYBR Green PCR Master Mix (TaKaRa Biotechnology). The qRT-PCR primers are listed in Table S1. The target mRNA expressions were normalized to *ACTB* or* Actb* expression. All reactions were processed on the Applied Biosystems 7500 Real-Time PCR System (Thermo Fisher Scientific, MA, USA). The test results were analyzed using the 2^-ΔΔCt^ method.

### ELISA Assay

Blood samples were collected and then serum was removed after centrifugation for 15 minutes at 1000 ×g. Plasma estrogen (E2) concentrations were measured using a double-antibody sandwich ELISA at room temperature as per the manufacturer's instructions (R&D Systems, Minneapolis, MN, USA). Absorbance was recorded at a dual-wavelength of 450/630 nm. Each plate also contained a standard control (coefficient of variation < 12%).

### Cells Culture Experiments

The monocytes cell line, U937, and the human endometrium stromal cell line, HESC, were purchased from the ATCC collection. HESC were pre-treated by RU486 (1 nM or 10 nM; Sigma, MA, USA) for 48h, then treated with or without PPD (40 μM; Sigma, MA, USA) for 48h, and the expression of related genes were detected by qRT-PCR. HESCs and DSCs were treated with or without PPD (40 µM) for 48 h, and the expression of related genes were detected by qRT-PCR. U937 cells were treated with 100 ng/mL phorbol-12-myristate 13-acetate (PMA, Sigma, MA, USA) for 24 h for differentiation and then they were co-cultured with PPD-pretreated HESCs for 48h. These U937 cells were collected to analyze the expression of IL-12, and IFN-γ by FCM.

### mRNA-seq

Total RNA was extracted from the samples by Trizol reagent (Invitrogen, CA, USA) separately. Gene expression analysis was conducted by mRNA-seq for the conditions described in the relevant figures. Quality of the total RNA was measured by the Agilent 2100 Bioanalyzer and samples with RNA integrity number (RIN) higher than 7 were used for sequencing. cDNA libraries were generated using the TruSeq Stranded mRNA Library Prep Kit (Illumina, Inc.) according to the manufacturer's instructions. Libraries were size selected using a 6% polyacrylamide gel and purified using the QIAQuick PCR Purification Kit (Qiagen GmbH). Purified libraries were normalized and pooled to create a double stranded cDNA library ready for sequencing. The samples were sequenced on the Illumina MiSeq to render 50 base pair single end reads. The sequencing library was constructed after high-quality RNA was quantified and then sequenced HiSeq X (Illumina, CA, USA) on a 150 bp paired-end run.

### Dif-Gene Analysis, Go Analysis, and Pathway Analysis

We applied DESeq2 algorithm 32 to filter the differentially expressed genes, after the significant analysis. The P-value and FDR analysis 33 were subjected to the following criteria: i) Fold Change>2 or < 0.5; ii), P-value<0.05, FDR<0.05. Gene ontology (GO) analysis 34 and pathway analysis have down as reported 35.

### Integration analysis of the Protein‑protein Interaction (PPI) Network and Predict sensory proteins of PPD

The related moleules were submitted to the STRING database (available online: http://string‑db.org) for PPI recognition. The PPI network is further visualized by using the software of Cytoscape 3.7.2. We used to predict potential cellular sensory proteins or target receptors via the Swiss Target Prediction platform (http://www.swisstargetprediction.ch/).

### Isolation and Purification of dNK cells

All subjects completed informed written consents forms for tissue collection, and the present study was approved by the Human Research Ethics Committee of Obstetrics and Gynecology Institute, Fudan University (Shanghai, China). Decidual tissues (n=19) were obtained from healthy women during early pregnancy (age, 23-35 years old; gestational age, 7-9 weeks) who underwent elective terminations for non‐medical reasons. The decidual tissues were digested and isolated as a previous procedure 36. Using this method, 98% of cells obtained were vimentin^+^ cytokeratin (CK)7^-^ DSCs and CD45^+^ DICs as previously reported 25. DICs were used for dNK cells isolation, using magnetic beads (Miltenyi Biotec, Bergisch Gladbach, Germany) for *in vitro* experiments. These NK cells were directly treated with PPD (10, 20 or 40 µM, Sigma, MA, USA) or vehicle control (0.1% DMSO, Sigma, MA, USA) for 48h, and the dNK cells were collected to analyze the expression of NKp30, Ki67, CD16, VEGF, TGF-β1, and CXCL-10 by FCM.

### *In vivo* X-ray Computed Microtomography (Micro-CT)

The isolated femurs were placed in a holder in the supine position. X-ray micro-computed tomographic scanning of the mice was performed using the Skyscan 1076 Scanner. The present research set the energy as 70 KVp and 100 µA with medium image resolution to obtain the best contrast between bone and soft tissues. The volume of interest (200 slices) for trabecular bone parameters was selected at the metaphyseal area located 1.5 mm below the lowest point of the epiphyseal growth plate of proximal tibia extending distally. To determine the cortical bone parameters, 100 slices were analyzed at the diaphyseal area located 2.5 mm from the metaphyseal area.

### Statistical Analysis

Each experiment was conducted at least three times independently. For data with only two groups, Student's t test was used. For data containing more than two groups and fit the normal distribution, an analysis of variance (ANOVA) test was used, followed by Tukey or Bonferroni test for t tests, and the results were presented as mean ± SEM. All analyses were conducted using the SPSS 20.0 Statistical Package for the Social Sciences software. p <0.05 was considered to indicate a statistically significant difference.

## Results

### PPD regulates hormones receptors expression and restricts the growth of ectopic lesions of EMs mice with impaired fertility and elevated inflammation

**Figure 1:**
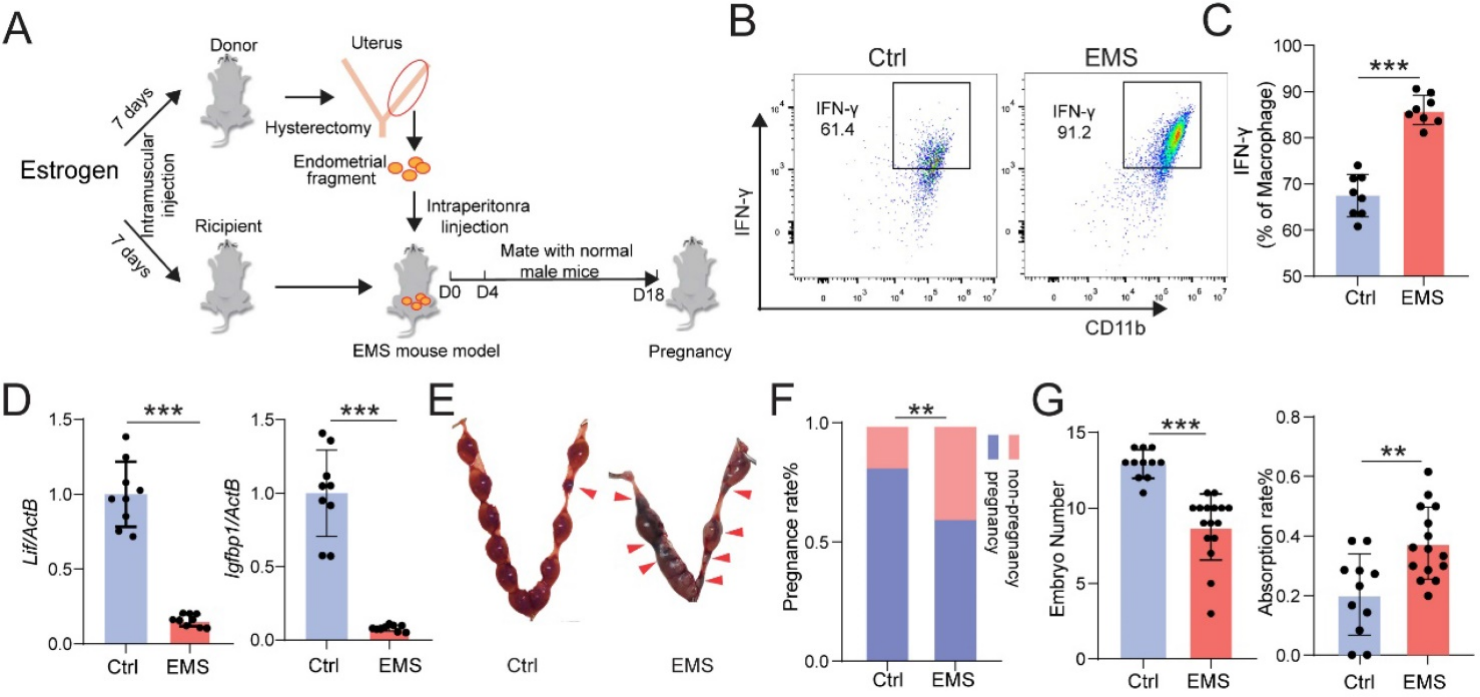
** EMs mice model with endometrial transplantation displays impaired fertility and elevated inflammation.** (A) On the day 4, peritoneal fluids and uterus of the normal BalB/C female mice (Ctrl) and / or EMs mice were collected, or the rest of Ctrl and / or EMs mice were randomly mated with healthy male adult mice, respectively, and then the data of pregnancy rate, the number of embryos, and the absorption rate were counted on day 18. (B&C) FCM was used to evaluate the expression of IFN-γ in CD45^+^F4/80^+^/CD11b^+^ Mφ macrophages of PF (n=8). (D) mRNA expression levels of *Lif* and *Igfbp1* in eutopic endometrium of Ctrl and EMs mice (n=10). (E) The pregnancy rates, number of implanted embryos and the embryo absorption rate of Ctrl mice (n=11) and EMs mice (n=16) at the gestation of day 13.5 were quantified in (F&G), and the absorption sites were indicated by the red triangle. Mean ± SEM, **p<0.01, ***p<0.001.

Chronic pelvic pain and infertility are the most common symptoms of EMs. We firstly constructed the EMs mice model by allotransplantation, flowing the procedure of Figure 1A. At Day 4, the expression level of interferon (IFN)-γ in Mφ of peritoneal fluid (PF) was measured by FCM, and the mRNA expression levels of* interleukin 6 family cytokine* (*Lif*) and* Insulin-like growth factor-binding protein 1* (*Igfbp1*) were measured by qRT-PCR. As shown, the levels of IFN-γ in CD45^+^F4/80^+^/CD11b^+^ Mφ were significantly increased (Fig. 1B and 1C), and the expression of *Lif* and *Igfbp1* were obviously downregulated (Fig. 1D) in uterine endometrium from mice with EMs. Additionally, these female mice were randomly mated with healthy male mice for another 14 days. At Day 18, we observed poor pregnancy outcomes in the EMs mice group, including lower pregnancy rate and embryo implantation numbers and higher embryo resorption rates, compared with the Ctrl group (Fig. 1E-G). These data suggest that the EMs mice model with endometrial allotransplantation can reflect the condition (i.e. elevated pelvic inflammation) *in vivo* and impaired fertility of endometriosis.

**Figure 2:**
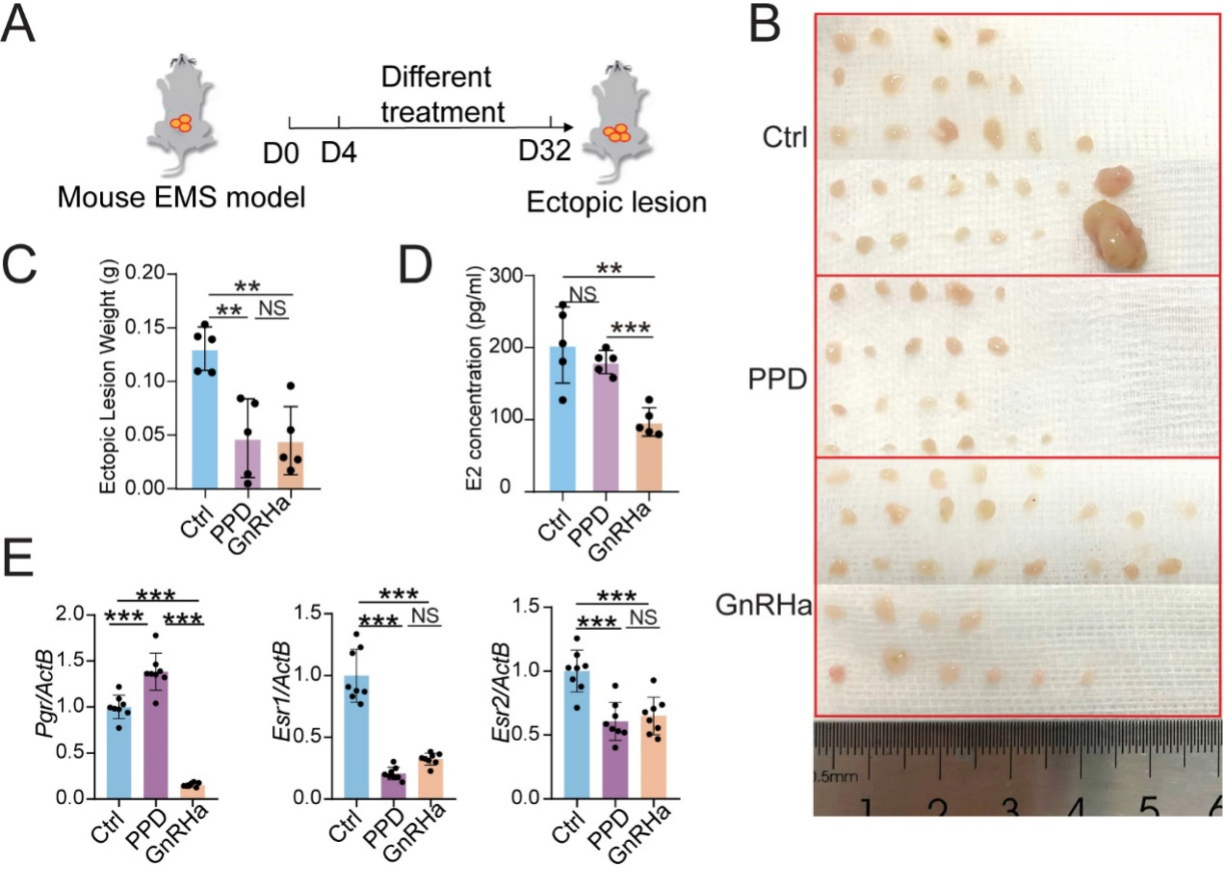
** PPD regulates hormones receptors expression and restricts the growth of ectopic lesions of EMs mice.** (A) The EMs model of BALB/C mice were intraperitoneally injected with control vehicle (5 % DMSO, every days), PPD (45 mg/kg, every 4 days), and GnRHa (0.5 µg, every day) from day 4 to day 32. (B-D) On day 32, the number (B) and weight of ectopic lesions (C) were analyzed. The serum concentrations of E2 were measured by ELISA (D) (n=5). (E) mRNA expression levels of* Pgr*, *Esr1* and *Esr2* in eutopic endometrium of EMs mice (n=8). Mean ± SEM, NS, no significant difference, **p<0.01, ***p<0.001.

To evaluate the potential therapeutic role of PPD in EMs, PPD (45 mg/Kg, every 4 days) or GnRHa (as a positive control, 0.5 μg, every day) was used to treat the EMs mice model, flowing the procedure of Figure 2A. Excitingly, the number and weight of ectopic lesions were diminished significantly in the PPD-treated group, as well as the GnRHa-treated group (Fig. 2B and 2C). More notedly, PPD did not affect the serum estrogen (E2) concentration, while GnRHa led to the down-regulation of E2 concentration dramatically as reported 37 (Fig. 2D). Interestedly, treatment with PPD resulted in up-regulation of *Progestogen receptor* (*Pgr*) and down-regulation of *Estrogen receptor 1* (*Esr1*) and *Esr2* (Fig. 2E).

### Target Acquisition of PPD

To further analyze the possible mechanism of PPD on endometrial receptivity, Swiss Target Prediction (http://swisstargetprediction.ch/) was carried out, and the data showed that there were 66 predicted intracellular sensory proteins or target reporters, including androgen receptor (AR), 11-beta-hydroxysteroid dehydrogenase 1 (HSD11B1), nuclear receptor ROR-gamma (RORC), ERα, and ERβ (Table 1).

### PPD up-regulates the expression of endometrial receptivity-related genes

**Figure 3:**
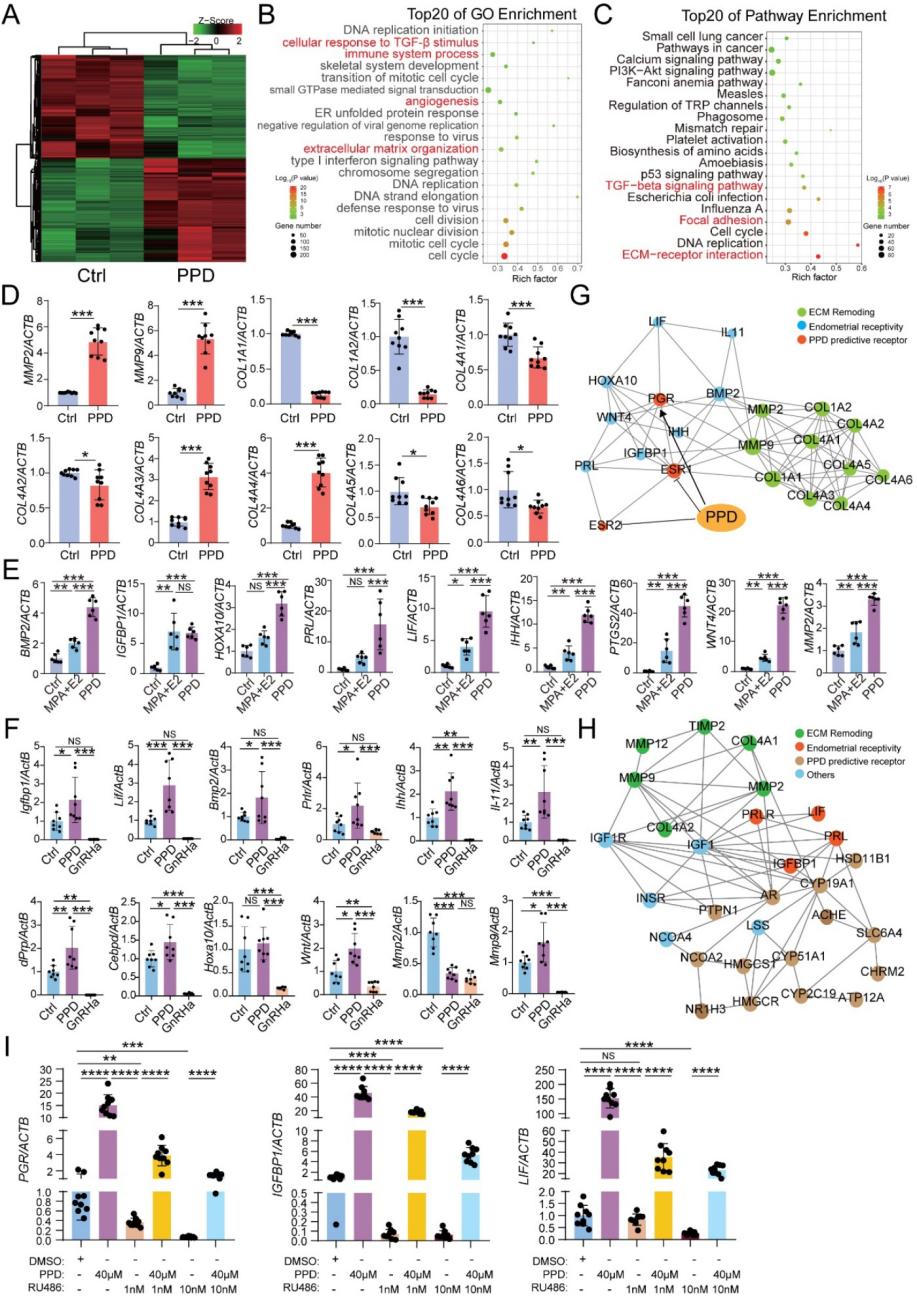
** PPD promotes the endometrial receptivity possibly in hormone receptor dependent and independent manners.** (A-C) mRNA-seq was performed to evaluate the differential expression genes in HESCs after treatment with or without PPD (40 µM) for 48 h. Gene Ontology (GO) enrichment and KEGG pathway enrichment analyzed have been shown. (D) qRT-PCR was used to evaluate the expressions of MMPs and collagens in HESCs after treatment with or without PPD (40 µM) for 48 h. (E) HESCs were treated by DMSO (1‰), or medroxyprogesterone acetate (MPA, 1 µM) combining with estradiol (E2, 1 nM), or PPD (40μM) for 48 h, and RT-PCR was used to measure the expression level of endometrial receptivity-related genes. (F) The mRNA level of endometrial receptivity-related genes in endometrium of EMs mice treated by control vehicle (5‰DMSO, every day), PPD (45 mg/kg, every 4 days), and GnRHa (0.5 µg, every day) (n=8). (G) PPI network of predicted sensory proteins of PPD (ESR1 and ESR2), PGR, endometrial receptivity-related molecules and collagens were obtained by STRING database and Cytoscape. (H) Top 15 predicted sensory proteins of PPD (get from SWISS Target Prediction website, showed in Table 1) involved in the regulation of endometrial receptivity and ECM remodeling, including AR, CYP19A1, HSD11B1, etc. (I) HESC were pre-treated with RU486 (1 nM or 10 nM) for 48h, then treated with or without PPD (40 µM) for 48h, or treated with DMSO (1 %) as control. Then, the mRNA level of *PGR*, *IGFBP1* and *LIF* were analyzed by RT-PCR. Mean ± SEM, NS, no significant difference, *p<0.05, **p<0.01, ***p<0.001, ****p<0.0001.

To explore the possible mechanism for PPD on the fertility of EMs, RNA-sequence was performed to evaluate the potential effects of PPD on human endometrial stromal cells line (HESCs). As shown, there were 2139 differential up-regulated genes (e.g., *MMPS*, *IGFBP1*, *LIF*, *PRLR*) and 2136 down-regulated genes (e.g., *COL1A1*, *COL1A2*, *COL4A1*, *ESR1*) in PPD-treated HESCs vs. Ctrl HESCs (Fig. 3A). The Top 20 of GO function and KEGG pathway enrichment analysis showed that the differential expressed genes were mainly involved in the cellular response to TGF-β stimulus, immune system process, angiogenesis, extracellular matrix-related pathways, focal adhesion and TGF-β signaling pathway to regulate inflammation, cell adhesion (Fig. 3B and 3C), and these biological processes were closed related to endometrial receptivity and embryo implantation. Compared with the Ctrl HESCs, PPD-treated HESCs expressed higher levels of MMPs, and lower levels of collagens (Fig. 3D). As a common inducer of decidualization, MPA plus 17β- estradiol (E2) significantly increased the expression of genes related to endometrial receptivity (e.g., *BMP2, IGFBP1, HOXA1, PRL, LIF, IHH, PTGS2, WNT4, MMP2*). Surprisingly, the stimulatory effects of PPD on endometrial receptivity was even more significant (Fig. 3E). And these effects were further confirmed in EMs mice models (Fig. 3F). In contrast, GnRHa significantly suppressed endometrial receptivity (Fig. 3F), suggesting that PPD may promote the endometrial receptivity of EMs.

The protein-protein interaction (PPI) network of predicted sensory proteins of PPD (ER-α and ER-β), progesterone receptor (PR), endometrial receptivity-related molecules and collagens were obtained by STRING database and Cytoscape (Fig. 3G), suggesting that PPD induces the expression of endometrial receptivity-related molecules and collagen possibly by binding and regulating the expression of ESR and or PGR. Additionally, other sensory proteins of PPD were predicted, and involved in the regulation of endometrial receptivity and extracellular matrix (ECM) remodeling, including AR, CYP19A1, HSD11B1, etc. (Fig. 3H). To explore the potential relationship between PPD and PR, we used RU486, a PR inhibitor, to inhibit the PR expression of HESC, and found that with the increase of RU486 concentration, the inhibition of PR was enhanced. Due to the decrease of PR expression induced by RU486, the promotion effect of PPD on the expression of genes related to endometrial receptivity, *IGFBP1* and *LIF*, was significantly weakened. These data suggest that PR should be a potential regulator for PPD to play a regulating role in remolding fertility (Fig. 3I).

### PPD alleviates pro-inflammatory cytokines production of macrophage in endometriotic milieu

**Figure 4:**
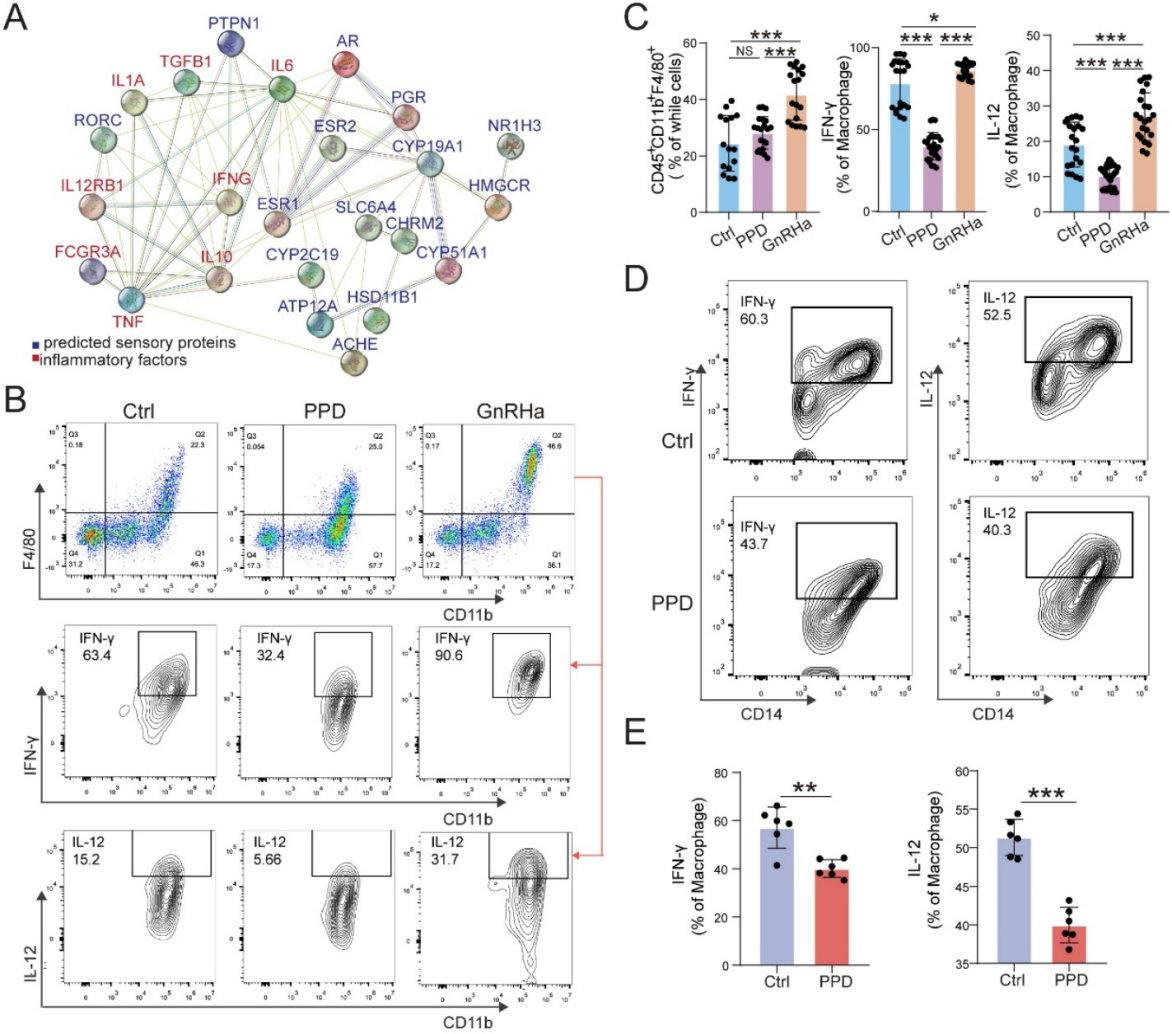
** PPD alleviates pro-inflammatory cytokines production of macrophage in endometriotic milieu.** (A) The PPI network of top 15 predicted sensory proteins of PPD and cytokines secreted by Mφ. (B&C) The expression level of inflammatory factors in CD45^+^F4/80^+^/CD11b^+^ Mφ of peritoneal fluids (PF) treated by the control (5 % DMSO, every day, n=15), PPD (45 mg/kg, every 4 days, n=18), and GnRHa (0.5 µg, every day, n=18) was evaluated by FCM. (D&E) FCM was used to measure the expression level of IFN-γ, and IL-12 in U937 cells co-cultured with or without PPD-pretreated (40 µM) HESCs for 48 h (n=6). Mean ± SEM, NS, no significant difference, *p<0.05, **p<0.01, ***p<0.001.

Owing to the key roles of macrophage in EMs, we further predict the possible role and PPI relationship between predicted sensory proteins and down-steam regulator (ESR1, ESR2 and PGR), and inflammatory cytokines by STRING, and the regulatory network was shown in Figure 4A. The data indicate that ESR1 and other sensory proteins (e.g., AR, HSD11B1, and RORC) should be an importance regulator for inflammatory cytokines. To further confirm this possible regulatory effect *in vivo*, we analyzed the levels of inflammatory cytokines and macrophage differentiation in PF from EMs mice models. As shown, PPD could significantly down-regulate the expression of IFN-γ and IL-12 in Mφ, compared with control and GnRHa groups (Fig. 4B and 4C). Similarly, the results in the co-culture model of PMA-pretreated U937 cells and PPD-pretreated HESC echoed these effects, as shown, the expression of IFN-γ and IL-12 were decreased (Fig. 4D and 4E).

### PPD prevents the risk of spontaneous abortion of EMs mice possibly by promoting decidualization and decidual NK cell residence and differentiation

**Figure 5:**
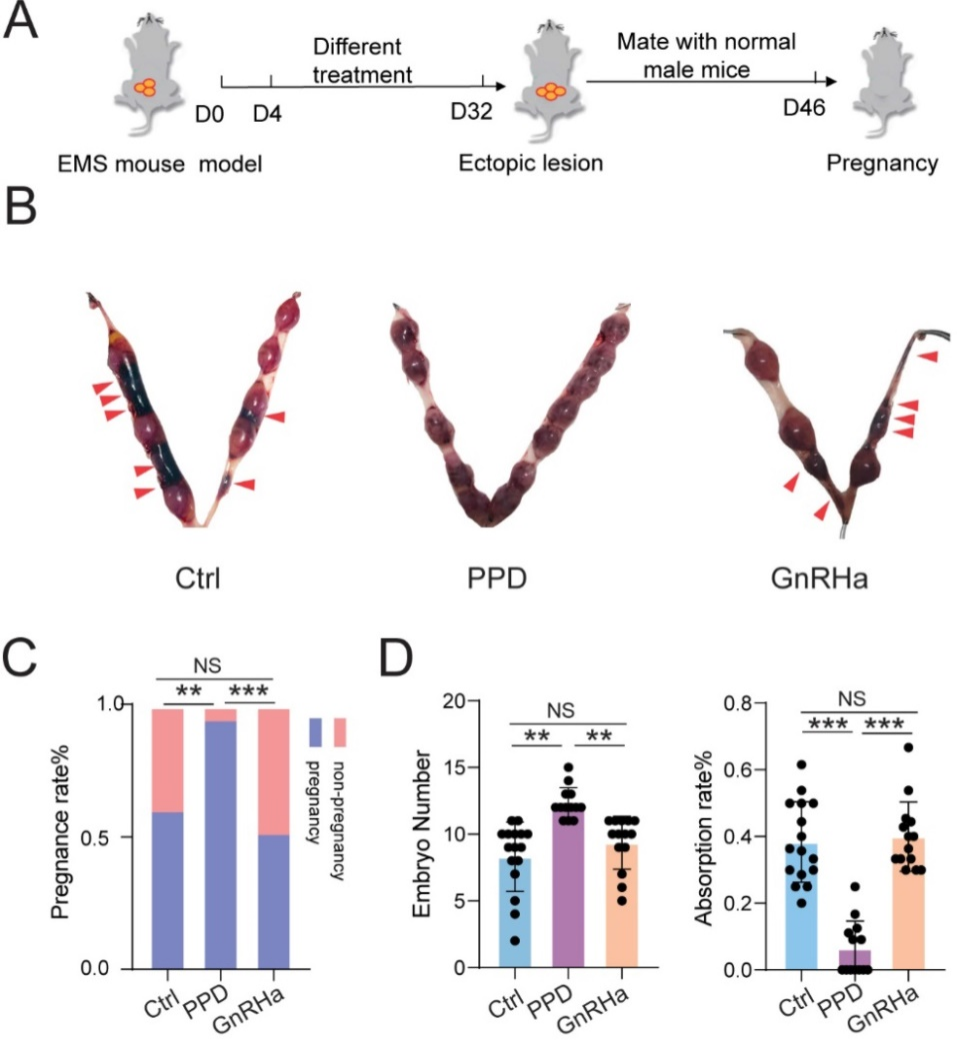
** PPD prevents the risk of spontaneous abortion of EMs mice.** (A) After 28 days of treatment with Ctrl (5 % DMSO, every days), PPD (45 mg/kg, every 4 days), and GnRHa (0.5 µg, every day), these female EMs mice were mated with healthy adult male mice. (B-D) The pregnancy rates, number of embryos implanted and the embryo absorption rate of the control group, the PPD group, and the GnRHa group at the gestation of day 13.5 were quantified in (C&D), and the absorption sites were indicated by the red triangle (n=16). Mean ± SEM, NS, no significant difference, **p<0.01, ***p<0.001.

As expected, subsequently, we observed that treatment with PPD significantly increased the pregnancy rate and embryo implantation numbers, and decreased the risk of embryo abortion of EMs mice (Fig. 5A-5D). However, GnRHa could not improve the pregnancy rate, embryo implantation numbers and embryo absorption rates (Fig. 5A-5D). These results indicate that PPD can lead to higher pregnancy rate, more embryos implantation, and lower embryo miscarriage of EMs mice models.

**Figure 6:**
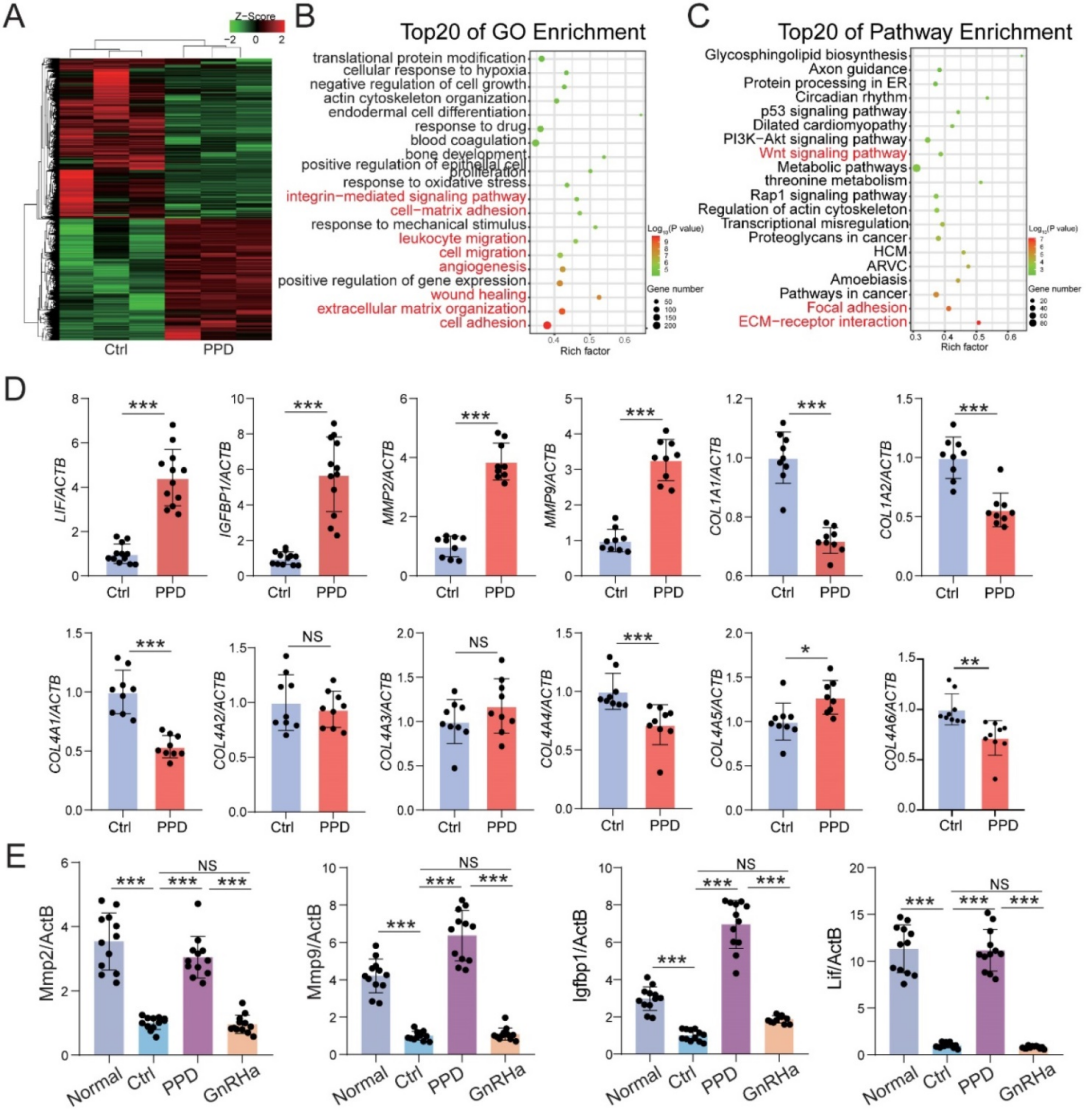
** PPD promotes decidualization by triggering multiple biological processes.** (A-C) mRNA-seq was performed to evaluate the differential expression genes in decidual stromal cells (DSCs) after treatment with or without PPD (40 µM) for 48 h. GO enrichment (B) and KEGG pathway enrichment (C) analyzed have been shown. (D) qRT-PCR was used to evaluate the expressions of* LIF*, *IGFBP1*, *MMP*s and collagens in DSCs after treatment with or without PPD (40 µM) for 48 h. (E) The mRNA expressions of* LIF*, *IGFBP1*, and *MMP*s in uterus of normal pregnancy mice or EMs pregnancy mice of the control group, the PPD-treated EMs pregnancy mice group, and the GnRHa-treated EMs pregnancy mice group (n=12). Mean ± SEM, NS, no significant difference, *p<0.05, **p<0.01, ***p<0.001.

To investigate the role and potential mechanism of PPD against EMs-related infertility and miscarriage, Ctrl and PPD-treated human decidual stromal cells (DSC) were collected and analyzed by RNA sequencing (Fig. 6A). As shown, there were 2553 up-regulated genes (e.g., decidualization-related genes, *LIF* and *IGFBP1*) and 3372 downregulated genes (e.g., ECM organization/remodeling-related genes, *MMP2*, *MMP9*,* COL1A1*, *COL1A2* and *COL4A1*) in the PPD-treated group, compared with the Ctrl group (Fig. 6A). The top 20 of GO function and KEGG pathway enrichment cluster displayed that PPD might be involved in the regulation of integrin-mediated signaling pathway, cell-matrix adhesion, leukocyte migration, angiogenesis, extracellular matrix-related pathways, and focal adhesion (Fig. 6B and 6C). Subsequently, the data of *in vitro* and *in vivo* experiments verified these findings, as shown, PPD significantly up-regulated the decidualization with up-regulation of *LIF*, *IGFBP1*, *MMPs* and or down-regulation of *Collages* in HESCs and pregnant uterus of EMs mice (Fig. 6D and 6E).

**Figure 7:**
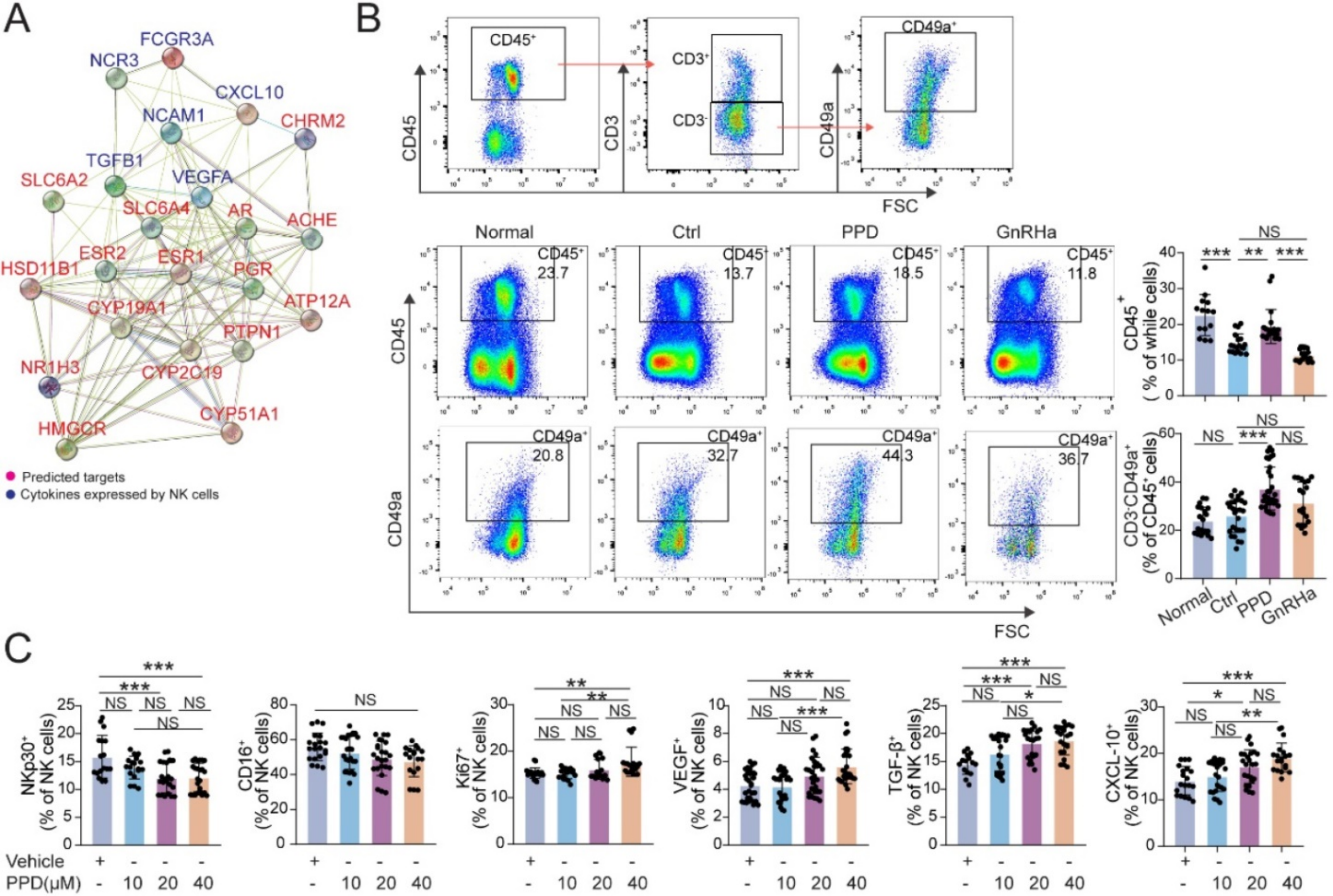
** PPD increases the residence, immune and angiogenic functions of decidual NK cells.** (A) The STRING tool was used to obtain PPI between predicted sensory proteins of PPD and cytokines produced by NK cells, and the PPI network was shown. (B) Flow cytometry gating strategy for identifying the CD45^+^ leukocytes and CD45^+^CD3^-^CD49a^+^dNK cells within the CD45^+^CD3^-^ gate, and quantitative analysis of the proportions of all leukocytes and dNK cells in the decidua of the Ctrl normal mice (n=20), the EMs mice treated by control vehicle (n=27), PPD (n=32), or GnRHa (n=19). (C) dNK cells separated for human decidual tissues, were treated with different concentrations of PPD (0, 10, 20, or 40 µM, n=18-30). Mean ± SEM, NS, no significant difference, *p<0.05, **p<0.01, ***p<0.001.

Owing to the important role of decidual NK cells in normal pregnancy 24, 26, 38, the PPI analysis between predicted sensory proteins (ESR and PGR) of PPD and cytotoxicity-related molecules (e.g., FCGR3A, NCR3) expressed by NK cells and the network was shown in Figure 7A. The results showed that ESR and PGR might be important intermediate regulators for NK cell (Fig. 7A). In EMs pregnant mice models, total CD45^+^ lymphocytes were significantly decreased in uterus and the decline became even more pronounced after GnRHa treatment (Fig. 7B). Notably, PPD markedly increased the percentage of total CD45^+^ lymphocytes and CD3^-^CD49a^+^NK cells in uterus of EMs pregnant mice (Fig. 7B). Further analysis showed that PPD could significantly down-regulate the expression cytotoxicity-related molecules (NKp30 and CD16), up-regulated the expression of Ki67, VEGF, TGF-β and CXCL10 of human decidual NK cells *in vitro*, especially at the concentration of 40 µM (Fig. 7C). These data suggest that PPD should promote the proliferation, decrease the cytotoxicity, promotes the angiogenesis and maternal-fetal immune tolerance.

### PPD does not cause bone loss with a good safety

**Figure 8:**
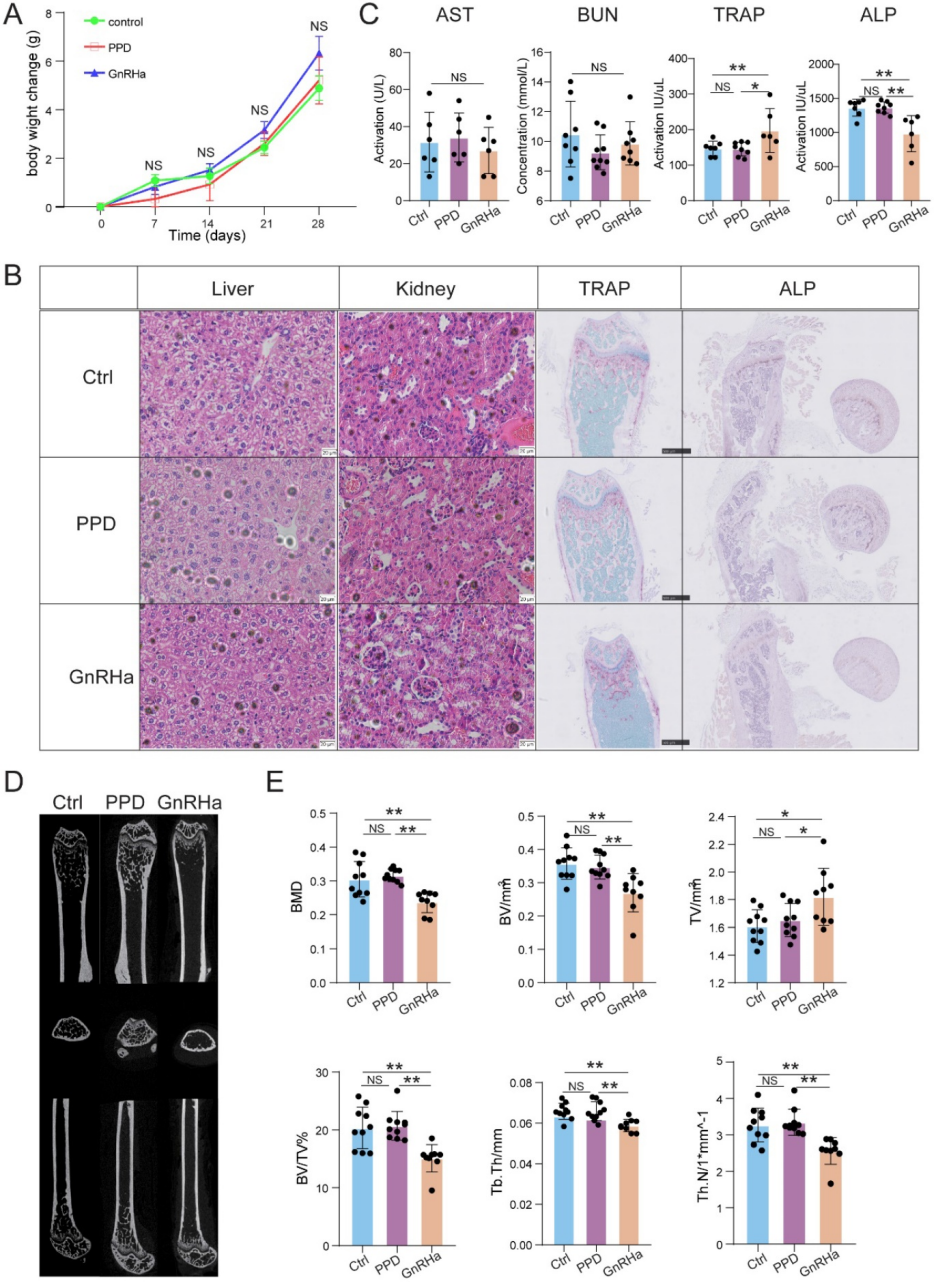
** PPD does not cause bone loss with a good safety.** (A) After 28 days for treatment, the bodyweight of the mice was recorded. (B) The H&E staining of the kidneys and livers, and the TRAP staining and ALP staining on mice femurs. (C) The serum biochemical index, and the levels of TRAP and ALP in the serum had been detected (n=6-9). (D) Three-dimensional micro-CT images of the trabecular microstructure of distal tibia metaphysis at the transaxial and axial view. (E) The BMD, BV, TV, BV/TV, trabecular thickness, and trabecular number of EMs mice in the control group, the PPD group and the GnRHa group were detected by Micro-CT (n=10). Mean ± SEM, NS, no significant difference, *p<0.05, **p<0.01.

To further evaluate the safety of PPD, the bodyweight of the mice was recorded, the function of liver, kidney, and bone evaluation were detected after 28 days for treatment. As a first-line drug for clinical use, GnRHa was also used as a comparison of evaluation. As shown, the bodyweight was no significant difference among the Ctrl, PPD and GnRHa groups (Fig. 8A). Besides, HE staining and serum biochemical index did not observe the dysfunctions of the kidney and liver among these groups (Fig. 8B and 8C). As a common side effect of GnRHa, we did observe the obstacle of bone remodeling in the GnRHa-treated group with high ratio of TRAP to ALP, which was also confirmed by TRAP staining and ALP staining on mouse femurs (Fig. 8B and 8C). According to the data of microCT, additionally, the bone mineral density (BMD), bone volume, tissue volume, trabecular thickness, and trabecular number in GnRHa-treated group at day 32 showed there was severe bone loss (Fig. 8D and 8E). However, we did not find similar results in the Ctrl and PPD-treated groups (Fig. 8B-8E). These results suggest that PPD should be more safety for treatment of endometriosis compared with GnRHa.

## Discussion

Surgical removal of lesions and hormonal medication, often with severe side effects and variable efficacy, are the most common therapies of EMs 18. But neither surgery nor drugs can help to reverse infertility of EMs patients effectively. Techniques of assisted reproduction consisting of superovulation with *in vitro* fertilization represent effective treatment alternatives that improve fertility in patients suffering from endometriosis. However, it still has the disadvantages of being expensive and not improving pregnancy loss. There is therefore an urgent need to find new therapy for EMs-related infertility and miscarriage and to restore and remodel their fertility. In current study, we found that PPD exerted anti-EMs activity and had the ability of fertility remodeling by ESR and PGR-mediated regulation of endometrial receptivity and inflammation response of peritoneal Mφ, and preventing pregnancy loss by increasing decidualization-associating genes expression, and promoting proliferation and function regulation dNK cells. Additionally, it almost no side effects on hepatotoxicity, nephrotoxicity and bone loss.

There are four main factors that affect the development of EMs, including interacting endocrine, pro-inflammatory, immunologic and proangiogenic processes 39. Over-expression of estrogen is required in the proliferation of endometriotic lesions. Progesterone exerts as an anti-estrogen effect in the endometrium, in the way by inducing 17β-hydroxysteroid dehydrogenase 2 (HSD17B2) to produce more estrone to weaken the effects of estrogen 40, 41. However, there is extremely low level of PGR in endometriotic tissue that led to progesterone resistance. This is the most important reason why progesterone has been ineffective for treatment of EMs. Here, we found that PPD could minimize the size and weight of ectopic lesions instant of changing the serum levels of E_2_ but though downregulating the expression of *ESR1*, *ESR2*, and upregulating *PGR* expression in eutopic endometrium.

The potential health effects of ginsenosides include immunomodulatory, anti-stress, anti-carcinogenic, anti-inflammatory, anti-allergic, anti-diabetic effects, and anti-hypertensive effects as well as anti-atherosclerotic and regulatory effects on blood pressure and metabolism 42, 43. The structure of PPD is similar to steroid hormones, and it may bind to nuclear receptors, such as AR, ERs, glucocorticoid receptor, and PR to act its pharmacological effects 44-46. Using RU486 blocked PR expression, PPD-induced endometrial receptivity genes' up-regulation was impaired, indicating that PR should be one of the potential regulator of PPD. Zhang and his colleagues 47 used fluorescence polarization assay to find that PPD could bind to human ER-α with moderate affinities, in other words, PPD acted as agonists of ER-α. The results of Swiss Target Prediction also suggested that AR, ER-α, ER-β and so on, were potential targets of PPD. Further analysis of PPI networks, *in vitro* and *in vivo* trials showed that PPD-mediated fertility improvement and remodeling of EMs should be dependent on the ERs, PR and other potential cellular sensory proteins. More efforts should be applied to explore the underlying mechanisms.

In EMs patients, the chronic pelvic inflammation contributes to the increased risk of infertility 48, owing to physically blocking the fallopian tubes, dysfunction of uterine tubes, decreasing receptivity of endometrium, and hindering development of the oocyte and embryo 9. During normal menstrual periods, endometrial stromal cells and glands will undergo periodic changes with changes in estrogen and progesterone. But overproduced estrogen and persisted progesterone resistance in EMs depart from this normal physiological process 49. The imbalance of estrogen and progesterone leads to not only inflammatory microenvironment, but also impaired endometrial receptivity 50. The expression of key implantation biomarkers, HOXA10, LIF, IGFBP1, among others, were seen to be disrupted in women with EMs 50, 51. In this study, after the administration of PPD, the decreased endometrial receptivity was reversed. The expressions of endometrial receptivity or decidualization-associated genes were increased significantly before and after embryos implanting. For example, the expressions of *MMP2* and *MMP9* were significantly increased, and the expressions of collagens were significantly decreased after treatment with PPD, both *in vivo* and *in vitro*. Additionally, PPD could diminish the percentage of peritoneal Mφ in the total cells of PF *in vivo*, and reduce the pro-inflammatory cytokines, IL-12 and IFN-γ produced by macrophages significantly. According to the PPI network, these effects were probably due to the binding to different cellular sensory proteins of PPD. More researches are needed to explore the underlying mechanism. As a result, pregnancy rate and embryo implantation numbers were improved markedly. Interestedly, the embryo resorption rates of EMs mice undergoing the PPD treatment were decreased, suggesting that PPD has a potential therapy value against pregnancy loss induced by EMs.

During the blastocyst implantation, many changes happened at the maternal-fetal interface, for incident, CD56^brigh^CD16^low^ NK cells were recruited by CXCR4 and CXCL12 secreted by embryonic trophoblast cells toa proper fetomaternal immune tolerance 52. Uterus NK cells (uNK) are the predominant leukocyte populations in endometrium and uNK increase and contribute to remodeling the uterine arteries during early pregnancy 53-55. Normally speaking, uNK cells have weakly cytotoxicity, unlike peripheral blood NK cells. Increased expressions of natural cytotoxicity receptors, like NKp 30, NKp40, and CD16 are related to many diseases, miscarriage and preeclampsia 56. Successful implantation and pregnancy establishment largely depend on uNK cells for producing and secreting VEGF, TGF-β, CXCL10, LIF, which lead to the maturation of endometrial blood vessels, trophoblast invasion and successful placental development 57, 58. In the present work, uNK cells from Ctrl EMs mice expressed higher CD16 and NKp30, compared with EMs mice treated by PPD. the population of dNK cells from the PPD group was increased, PPD could induce dNK cells to differentiate in favor of embryo implantation, owing to higher expression of Ki67, VEGF, TGF-β, and CXCL10. The PPI network between cytokines produced by uNK cells and top 15 PPD predicted cellular sensory proteins show that ESR1, ESR2, and PGR were the center of this regulatory network. However, the molecular mechanism is needed to further research.

GnRHa used to be the first-line treatment, working by substantially suppressing systemic estrogen levels. The side effects of menopause, including bone loss, cannot be ignored 49. Here, the EMs mice treated by GnRHa 0.5 ug/d*28 days have obvious symptoms of osteoporosis, with increasing number of osteoclasts, decreasing number of osteoblasts, and the result of Micro-CT showed there was an increase of bone loss. These side effects did not show in the PPD-treated EMs mice. In addition, no abnormalities were observed in the H&E staining and biochemical function testing of livers and kidneys. Treatment strategy of PPD in EMs seems to be more safety than GnRHa. Certainly, more researches are needed in the future.

**Figure 9:**
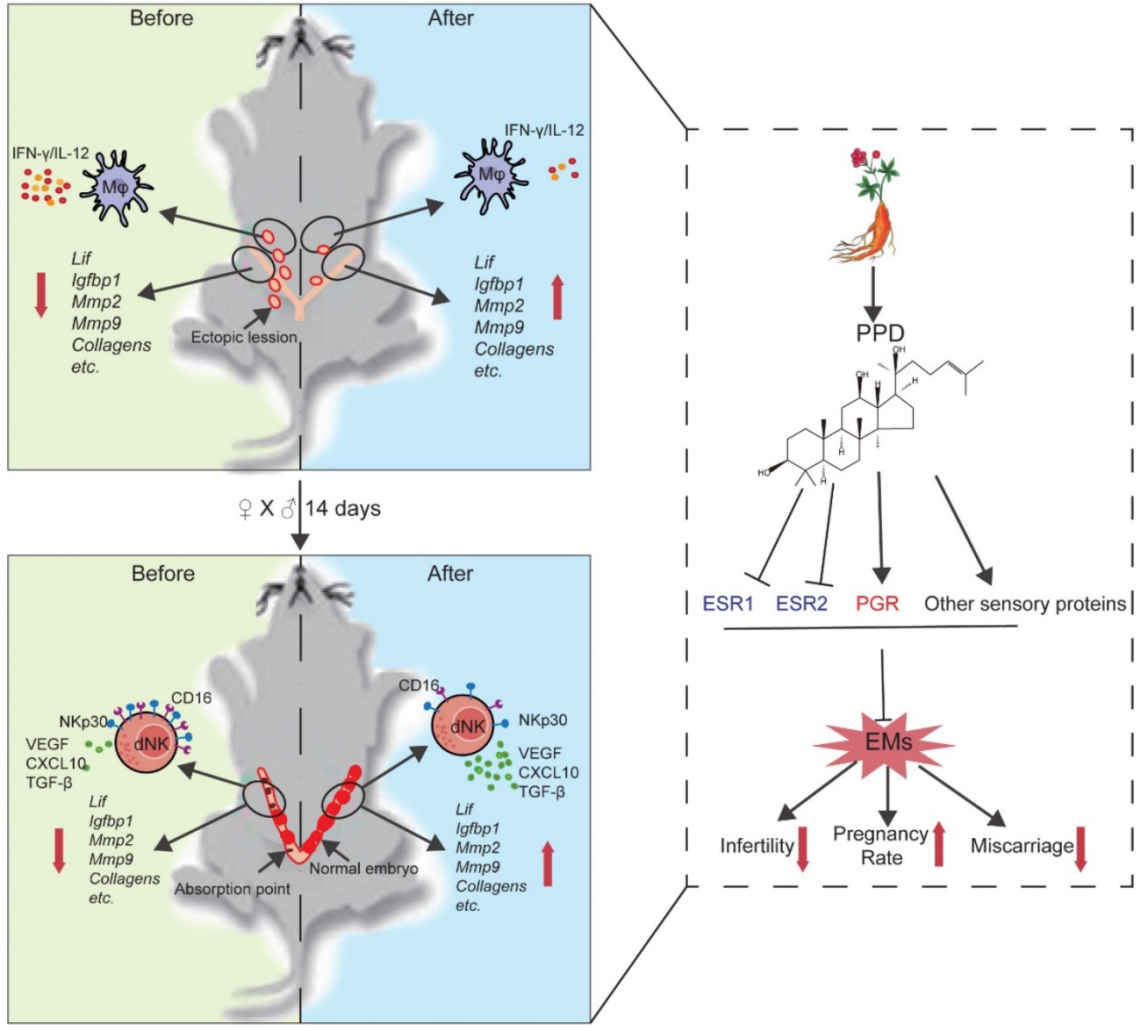
** Schematic roles of PPD in remodeling fertility and preventing the risk of spontaneous abortion of EMs.** Patients with endometriosis (EMs) have increased risks for infertility and spontaneous abortion. As one of two metabolites of ginsenoside (the main components extracted from ginseng), Protopanaxadiol (PPD) significantly up-regulates endometrial receptivity-related molecules (e.g., *Lif, Igfbp1, Mmps, collagens*) and restricts pelvic inflammatory response (low levels of IL-12 and IFN-γ) of macrophage, and further remodel and improve the fertility of EMs mice (upper part of figure). Additionally, PPD increases the expression of decidualization-related genes (e.g., *Lif, Igfbp1, Mmps*) and Collagens, and promotes the proliferation, residence, immune tolerance and anagogic functions of decidual NK cells (low levels of CD16 and NKp30, high levels of Ki67, VEGF, TGF-β and CXCL10) in pregnant EMs mice, and further triggers decidualization, decidual NK cell-mediated maternal-fetal immune tolerance and angiogenesis, preventing pregnant EMs mice from miscarriage. Mechanically, these effects should be dependent on ESRs, PGR and other sensory proteins. Therefore, the potential therapeutic value of PPD in EMs-related infertility and miscarriage should be emphasized due to high efficiency and low side effects.

In summary, as shown in Fig. 9, we have proposed a new strategy for the treatment of EMs and EMs-related infertility and miscarriage. From multiple angles, PPD is primary confirmed as a safe and effective compound for the treatment of EMs, including reducing ectopic foci, suppressing inflammation response of peritoneal Mφ, promoting endometrial receptivity and decidualization, and increasing the proportion, tolerance and pro-angiogenesis phenotypes of dNK cells. These effects should be dependent on hormones receptors or sensory proteins (ESRs and PGR), and other cellular sensory proteins. More efforts are needed to find out the underlying molecular mechanism.

## Supplementary Material

Supplementary table S1.

## Figures and Tables

**Table 1:** The information of potential cellular sensory proteins or targeted receptors of PPD

ID	Uniprot ID	Gene symbol	Protein name	Target Class	Known actives (3D/2D)
1	P18031	PTPN1	Protein-tyrosine phosphatase 1B	Phosphatase	85/49
2	P33261	CYP2C19	Cytochrome P450 2C19	Cytochrome P450	11/2
3	P10275	AR	Androgen Receptor	Nuclear receptor	44/77
4	P11511	CYP19A1	Cytochrome P450 19A1	Cytochrome P450	5/9
5	P08172	CHRM2	Muscarinic acetylcholine receptor M2	Family A G protein-coupled receptor	42/2
6	P23975	SLC6A2	Norepinephrine transporter	Electrochemical transporter	25/2
7	P31645	SLC6A4	Serotonin transporter	Electrochemical transporter	164/5
8	Q13133	NR1H3	LXR-alpha	Nuclear receptor	15/21
9	P22303	ACHE	Acetylcholinesterase	Hydrolase	45/2
10	Q16850	CYP51A1	Cytochrome P450 51 (by homology)	Cytochrome P450	10/3
11	P04035	HMGCR	HMG-CoA reductase	Oxidoreductase	101/3
12	P03372	ESR1	Estrogen receptor alpha	Nuclear receptor	17/38
13	P28845	HSD11B1	11-beta-hydroxysteroid dehydrogenase 1	Enzyme	289/21
14	P54707	ATP12A	Potassium-transporting ATPase alpha chain 2	Primary active transporter	7/3
15	P51449	RORC	Nuclear receptor ROR-gamma	Nuclear receptor	23/9
16	Q12772	SREBF2	Sterol regulatory element-binding protein 2	Unclassified protein	0/1
17	P05093	CYP17A1	Cytochrome P450 17A1	Cytochrome P450	0/28
18	Q9Y233	PDE10A	Phosphodiesterase 10A	Phosphodiesterase	326/0
19	P11473	VDR	Vitamin D receptor	Nuclear receptor	72/46
20	Q9UHC9	NPC1L1	Niemann-Pick C1-like protein 1	Other membrane protein	15/11
21	P16662	UGT2B7	UDP-glucuronosyltransferase 2B7	Enzyme	0/22
22	O00748	CES2	Carboxylesterase 2	Enzyme	8/7
23	Q14994	NR1I3	Nuclear receptor subfamily 1 group I member 3	Nuclear receptor	1/2
24	P06276	BCHE	Butyrylcholinesterase	Hydrolase	19/2
25	P32246	CCR1	C-C chemokine receptor type 1	Family A G protein-coupled receptor	125/0
26	Q9UM73	ALK	ALK tyrosine kinase receptor	Kinase	98/0
27	P11712	CYP2C9	Cytochrome P450 2C9	Cytochrome P450	10/0
28	P08684	CYP3A4	Cytochrome P450 3A4	Cytochrome P450	12/0
29	P35354	PTGS2	Cyclooxygenase-2	Oxidoreductase	54/3
30	P11474	ESRRA	Estrogen-related receptor alpha	Nuclear receptor	2/0
31	O95718	ESRRB	Estrogen-related receptor beta	Nuclear receptor	2/0
32	P04278	SHBG	Testis-specific androgen-binding protein	Secreted protein	1/41
33	P25116	F2R	Proteinase-activated receptor 1	Family A G protein-coupled receptor	36/0
34	Q16549	PCSK7	Subtilisin/kexin type 7	Protease	1/0
35	Q16602	CALCRL	Calcitonin gene-related peptide type 1 receptor	Family B G protein-coupled receptor	23/0
36	P40189	IL6ST	Interleukin-6 receptor subunit beta	Membrane receptor	2/0
37	P37288	AVPR1A	Vasopressin V1a receptor	Family A G protein-coupled receptor	30/0
38	P06213	INSR	Insulin receptor	Kinase	37/0
39	O00408	PDE2A	Phosphodiesterase 2A	Phosphodiesterase	44/0
40	Q07343	PDE4B	Phosphodiesterase 4B	Phosphodiesterase	47/0
41	O75469	NR1I2	Pregnane X receptor	Nuclear receptor	5 /0
42	P07900	HSP90AA1	Heat shock protein HSP 90-alpha	Other cytosolic protein	67 /0
43	P49327	FASN	Fatty acid synthase	Transferase	102 /0
44	Q16539	MAPK14	MAP kinase p38 alpha	Kinase	238 /0
45	Q13882	PTK6	Tyrosine-protein kinase BRK	Kinase	14 /0
46	O00329	PIK3CD	PI3-kinase p110-delta subunit	Enzyme	48 /0
47	P42338	PIK3CB	PI3-kinase p110-beta subunit	Enzyme	54 /0
48	P48736	PIK3CG	PI3-kinase p110-gamma subunit	Enzyme	40 /0
49	P42336	PIK3CA	PI3-kinase p110-alpha subunit	Enzyme	314 /0
50	Q00987	MDM2	p53-binding protein Mdm-2	Other nuclear protein	135 /0
51	Q9Y5P4	COL4A3BP	Collagen type IV alpha-3-binding protein	Unclassified protein	1 /0
52	P54760	EPHB4	Ephrin receptor	Kinase	39 /0
53	Q92731	ESR2	Estrogen receptor beta	Nuclear receptor	14 / 46
54	P49810 Q9NZ42 Q92542 Q96BI3 P49768 Q8WW43	PSEN2 PSENEN NCSTN APH1A PSEN1 APH1B	Gamma-secretase	Protease	83 /0
55	P45983	MAPK8	c-Jun N-terminal kinase 1	Kinase	102 /0
56	P17655	CAPN2	Calpain 2	Protease	24 /0
57	P24385 P11802	CCND1 CDK4	Cyclin-dependent kinase 4/cyclin D1	Kinase	31 /0
58	O96020 P24941 P24864	CCNE2 CDK2 CCNE1	Cyclin-dependent kinase 2/cyclin E	Other cytosolic protein	29 /0
59	P06737	PYGL	Liver glycogen phosphorylase	Enzyme	23 /0
60	P48039	MTNR1A	Melatonin receptor 1A	Family A G protein-coupled receptor	5 /0
61	P49286	MTNR1B	Melatonin receptor 1B	Family A G protein-coupled receptor	6 /0
62	P42345	MTOR	Serine/threonine-protein kinase mTOR	Kinase	376 /0
63	P42330	AKR1C3	Aldo-keto-reductase family 1 member C3	Enzyme	8 /0
64	P41146	OPRL1	Nociceptin receptor	Family A G protein-coupled receptor	53 /0
65	P35372	OPRM1	Mu opioid receptor	Family A G protein-coupled receptor	191 /0
66	P41145	OPRK1	Kappa Opioid receptor	Family A G protein-coupled receptor	137 /0

The targets of PPD were predicted by Swiss Target Prediction (http://swisstargetprediction.ch/) were shown (probability >0.1).

## Abbreviations

ALP: alkaline phosphatase
AR: androgen receptor
AST: aspartate aminotransferase
BUN: blood urea nitrogen
CXCL10: CXC chemokine ligand 10
dNK: decidual NK
E2: estradiol
ECM: extracellular matrix
ELISA: enzyme linked immunosorbent assay
EMs: endometriosis
ER: estrogen receptor
FCM: flow cytometry
GnRHa: gonadotropin-releasing hormone agonists
H&E: Hematoxylin-Eosin
HESC: human endometrium stromal cell line
HSD11B1: 11-beta-hydroxysteroid dehydrogenase 1
HSD17B2: 17β-hydroxysteroid dehydrogenase 2
IFN-γ: interferon-γ
IGFBP1: insulin-like growth factor-binding protein 1
IL-12: interleukin-12
LIF: interleukin 6 family cytokine
Mφ: macrophages
PGE2: Prostaglandin E2
PMA: phorbol-12-myristate 13-acetate
PPD: Protopanaxadiol
PR: progesterone receptor
RORC: nuclear receptor ROR-gamma
TGF-β: transforming growth factor-β
TRAP: tartrate resistant acid phosphatase
uNK: uterus natural killer
VEGF: vascular endothelial growth factor
