# Supplementary material for: Protopanaxadiol improves endometriosis associated infertility and miscarriage in sex hormones receptors-dependent and independent manners
Source: Int J Biol Sci. 2021 May 5;17(8):1878–94. doi: 10.7150/ijbs.58657 (PMC8193274; doi:10.7150/ijbs.58657)
Supplement: Supplementary file 1 — Supplementary table S1. [file ijbsv17p1878s1.pdf]

Table S1. Gene primers for qRT-PCR

ACTB	Forward	GCCGACAGGATGCAGAAGGAGATCA
	Reverse	AAGCATTTGCGGTGGACGATGGA
BMP2	Forward	TGACGAGGTCCTGAGCGAGTTC
	Reverse	TGAGTGCCTGCGATACAGGTCTAG
COL1A1	Forward	TGGCAAAGAAGGCGGCAAAGG
	Reverse	AGGAGCACCAGCAGGACCATC
COL1A2	Forward	CCGTGGCAGTGATGGAAGTGTG
	Reverse	GCAGGACCAGCGTTACCAACAG
COL4A1	Forward	TCTGGCTGTGGCAAATGTGACTG
	Reverse	TTGTCCTGGTGGTCCCTGTGG
COL4A2	Forward	TGAACCTGGAGAGCCTGGATTGG
	Reverse	GTCCTCTGTTGCCTTGCTGTCC
COL4A3	Forward	ACCTGGCACACCTGGAGAACC
	Reverse	CATCCTTGCCTGGCTTACCTCTTG
COL4A4	Forward	TGAGAGGACCAGAAGGAGCCATG
	Reverse	GGAGACCCAGGGACGCCATC
COL4A5	Forward	TTGGCTGGCAACTGTAGATGTGTC
	Reverse	CGTGTCCTCAAGTCTCCTGCTTTC
COL4A6	Forward	GGTTGTGGCTGCTCCTGGTTAC
	Reverse	TCGTCCTCTCGCTCCTTTCTCAG
HOXA10	Forward	CTCGCCCATAGACCTGTGG
	Reverse	GTTCTGCGCGAAAGAGCAC
IGFBP1	Forward	CGAAGGCTCTCCATGTCACCA
	Reverse	TGTCTCCTGTGCCTTGGCTAAAC
LIF	Forward	CCAACGTGACGGACTTCCC
	Reverse	TACACGACTATGCGGTACAGC
IHH	Forward	AACTCGCTGGCTATCTCGGT
	Reverse	GCCCTCATAATGCAGGGACT
PRL	Forward	AAGCTGTAGAGATTGAGGAGCAAAC
	Reverse	TCAGGATGAACCTGGCTGACTA
PTGS2	Forward	TCTCCTGCCTACTGGAAGCC
	Reverse	CTAGTCCGGAGCGGGAAGAA
MMP2	Forward	GCCTCTCCTGACATTGACCTTGG
	Reverse	CACCACGGATCTGAGCGATGC
WNT4	Forward	GCTCTGACAACATCGCCTAC
	Reverse	GCCAGCACGTCTTTAC
Actb	Forward	GTGACGTTGACATCCGTAAAGA
	Reverse	GCCGGACTCATCGTACTCC
Bmp2	Forward	GAATGACTGGATCGTGGCACCTC
	Reverse	GGCATGGTTAGTGGAGTTCAGGTG
Cebpd	Forward	TCCACGACTCCTGCCATGTACG
	Reverse	GCTGTTGAAGAGGTCGGCGAAG

dPrp	Forward	CCTCATCACGTCTATACATGCG
	Reverse	GCTGCATCAATTCCTGAATGTCA
Esr1	Forward	CCCTCCCGCCTTCTACAGGTC
	Reverse	ACGGCACAGTAGCGAGTCTCC
Esr2	Forward	GCGACGACGGCACGGTTC
	Reverse	CTGCTTCCTGGCTTGCGGTAG
Hoxa10	Forward	GGCAGTTCCAAAGGCGAAAAT
	Reverse	GTCTGGTGCTTCGTGTAAGGG
Igfbp1	Forward	CCGCCACGAGCACCTTGTTC
	Reverse	AGCTGCTCCTCTGTCATCTCTGG
Ihh	Forward	CTCAGACCGTGACCGAAATAAG
	Reverse	CCTTGGACTCGTAATACACCCAG
Il-11	Forward	GTGCTGACAAGGCTTCGAGTAGAC
	Reverse	CAAGGCCAGGCGAGACATCAAG
Lif	Forward	GCCTCCAGGTCAAGCTCAATGC
	Reverse	ACACGGTACTTGTTGCACAGACG
Mmp2	Forward	TGGCACCACCGAGGACTATGAC
	Reverse	ACACCACACCTTGCCATCGTTG
Mmp9	Forward	GCAGAGGCATACTTGTACCG
	Reverse	TGATGTTATGATGGTCCCACTTG
Pgr	Forward	ATAGACAAGTCTCAGTGGAAAG
	Reverse	TCTGTTGTGTTGTACCTGGTT
Prlr	Forward	TAGATGGGGCCAGGAGAAATC
	Reverse	ACTGGAGAATAGAACACCAGAGC
Wnt4	Forward	AGACGTGCGAGAAACTCAAAG
	Reverse	GGAACTGGTATTGGCACTCCT
